# Multiple telescoping flow diverter technique in endovascular treatment of a vertebrobasilar dissecting aneurysm: case report

**DOI:** 10.3389/fneur.2023.1218154

**Published:** 2023-07-13

**Authors:** Ming-Yi Wang, Yong-Sheng Liu, Xiang-Bo An, Tao Pan, Feng Wang

**Affiliations:** Department of Intervention Therapy, First Affiliated Hospital of Dalian Medical University, Dalian, China

**Keywords:** flow diverter, telescoping technique, vertebrobasilar, dissecting, aneurysm

## Abstract

A 64-year-old man presented with headache and dizziness. A vertebrobasilar dissecting aneurysm was identified via computed tomography angiography and high resolution magnetic resonance imaging. Perioperatively, standard oral dual antiplatelet drugs were given. Two flow diverters were telespcoped for endovascular treatment of the aneurysm. Postoperatively, there were no signs of cerebral infarction and no new symptoms. At the 6-month follow-up, digital subtraction angiography showed that the aneurysm was almost completely occluded, with no other complications. This case serves as a reference for using the multiple telescoping flow diverter technique to treat vertebrobasilar dissecting aneurysm.

## Introduction

Surgical treatment of complex intracranial aneurysm is problematic, and endovascular treatment has high rates of complications and recurrence. Using a flow diverter (FD) makes treating this kind of aneurysm simpler and safer (1). The advantages of a flow diverter include low porosity and high metal coverage rates. Some studies, however, report that overlapping flow diverters increase the risks of the endovascular treatment and do not benefit the aneurysm cure rate (2).

It remains inevitable that in cases with large or giant aneurysms with extremely wide necks, or longer spindle-shaped aneurysms, two or more flow diverters must be inserted, through a telescoping technique. Studies that have reported the efficacy and safety of telescoping flow diverters in complex intracranial aneurysms treatment are limited (3, 4).

Here we report the case of a vertebrobasilar dissecting aneurysm treated with a multiple telescoping flow diverter technique, which may serve as a reference for the procedure.

## Case presentation

A 64-year-old man visited a local hospital due to headache and dizziness. The head computed tomography angiogram (CTA) showed an aneurysm, and he was referred to our hospital in February 2022.

**Figure 1:**
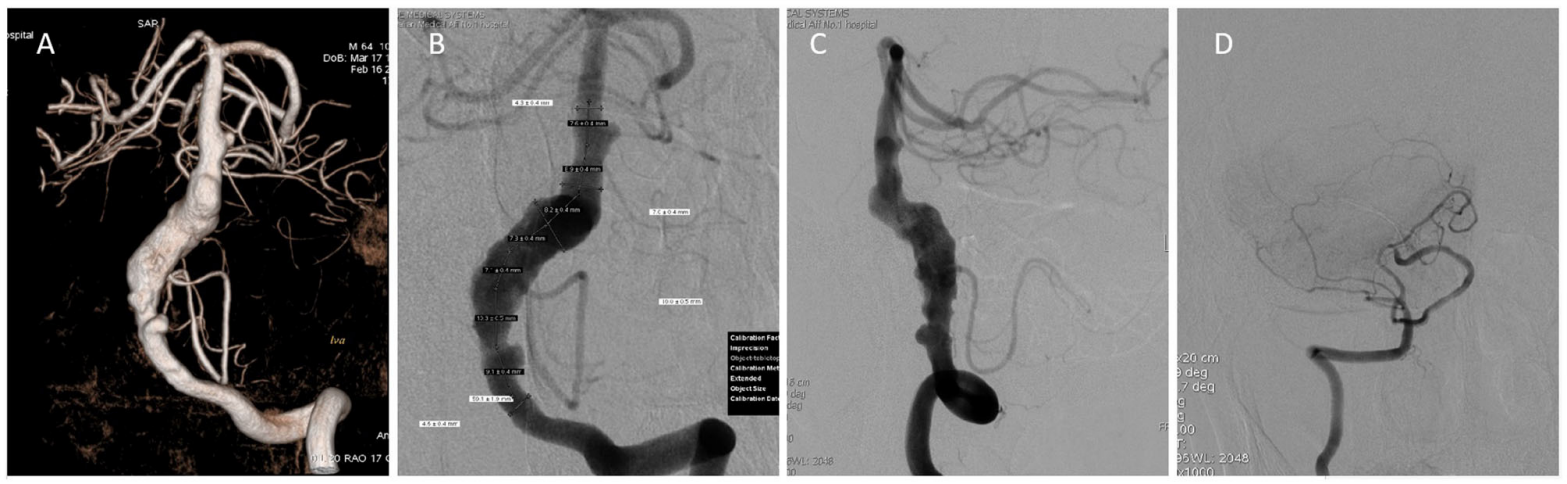
Preoperative angiography. **(A)** Three-dimensional angiography reveals a vertebrobasilar aneurysm. **(B)** Anteroposterior angiography measurement data shows the maximum outer diameter and the length of the aneurysm. **(C)** Lateral angiography reveals a vertebrobasilar aneurysm. **(D)** Anteroposterior angiography shows a slender right vertebral artery and occulded V4 segment.

**Figure 2:**
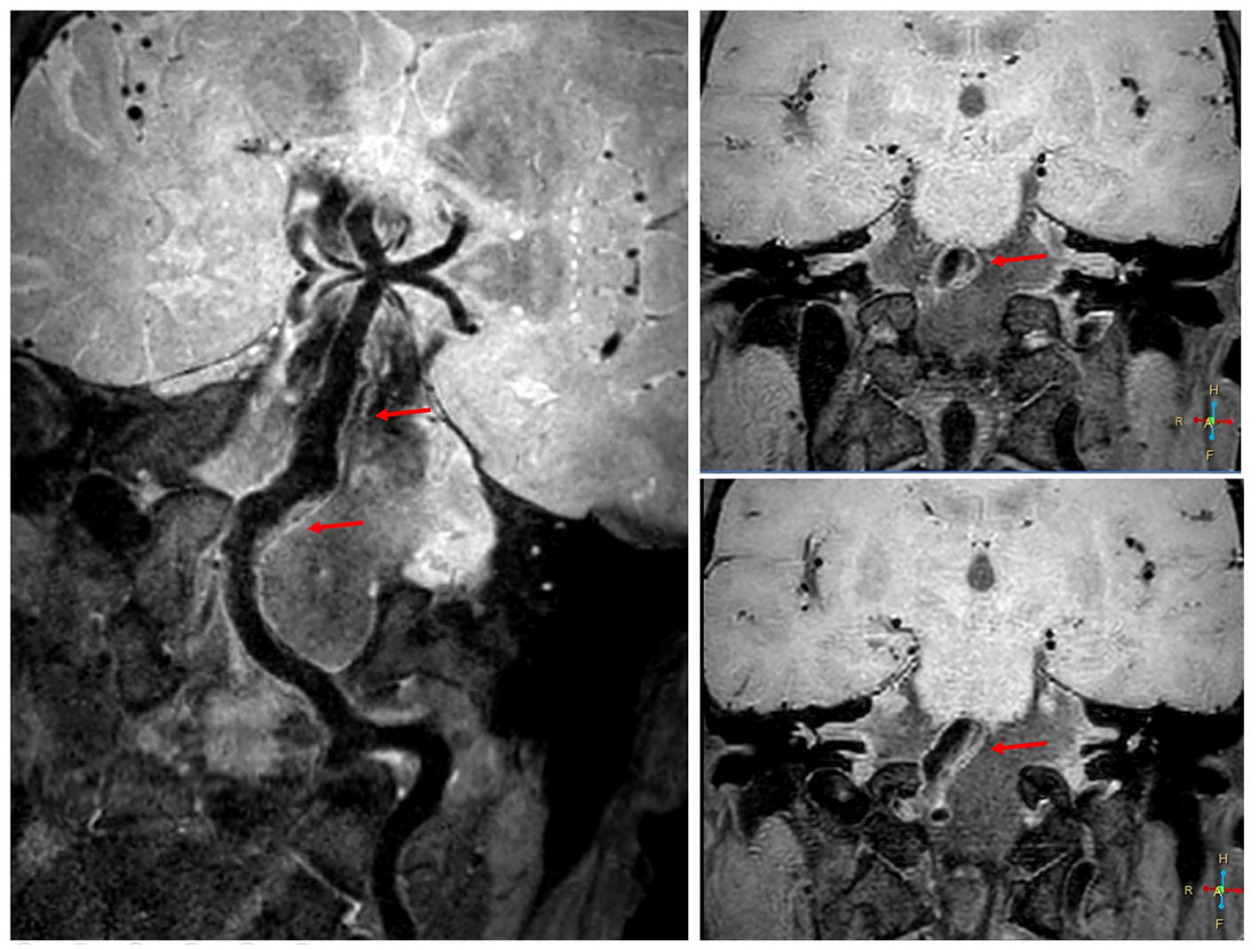
High resolution magnetic resonance imaging before the operation. High resolution magnetic resonance imaging reveals a vertebrobasilar dissecting aneurysm (red arrow).

The patient had a family history of subarachnoid hemorrhage. Digital subtraction angiography revealed a fusiform aneurysm at the left vertebral artery V4 segment to the proximal segment of the basilar artery, with a maximum diameter and length of about 10 and 60 mm, respectively; the right vertebral artery was slender, and the right vertebral artery V4 segment was occluded (Figure 1). High resolution magnetic resonance imaging confirmed that the lesion was vertebrobasilar dissecting aneurysm (Figure 2). Considering the characteristics of the aneurysm, we decided to use a flow diverter for the treatment. Because of the large area of the lesion, a single flow diverter could not completely cover the aneurysm, so two flow diverters were implanted via a telescoping technique.

The patient was administered oral antiplatelet drugs (aspirin 100 mg, and clopidogrel 75 mg, 1×/d) 5 days before the procedure. After 5 days, thromboelastography was performed. The arachidonic acid and adenosine diphosphate inhibition rates were 82 and 48%, respectively. Under general anesthesia, a 6F 90-cm long sheath (Cook, USA) was placed in the left subclavian artery, a 5F 115-cm Navien catheter (Medtronic, USA) was placed in the V3 segment of the left vertebral artery, and a microcatheter (Shanghai MicroPort, China) was inserted into the parent artery.

**Figure 3:**
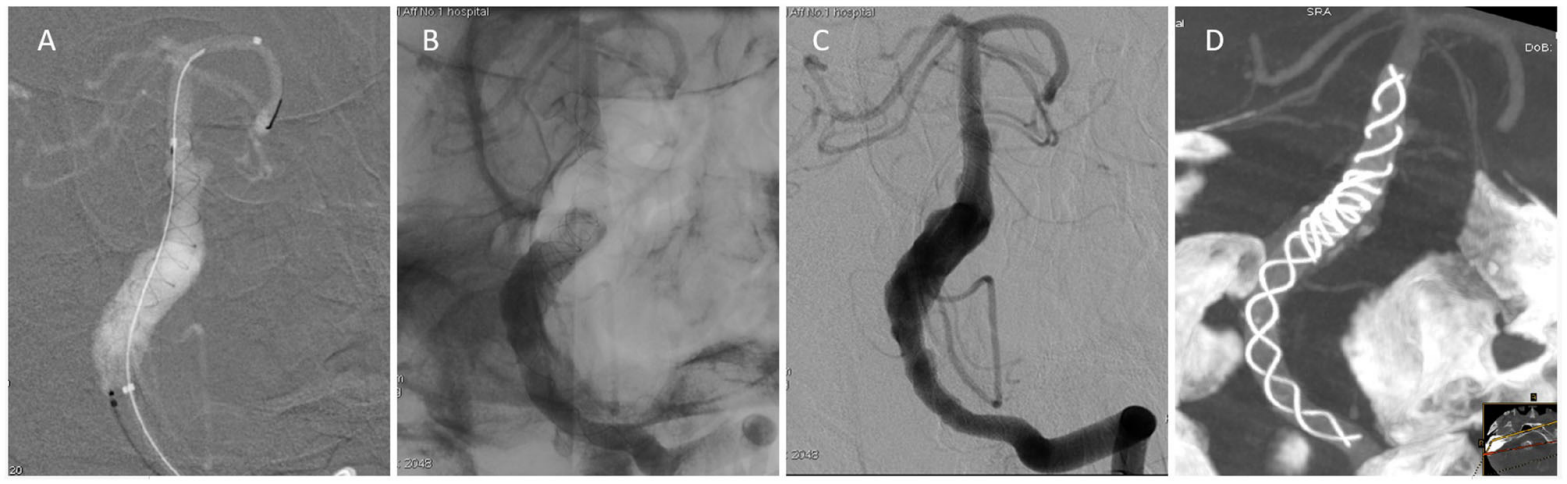
Angiography during the operation. **(A)** The first flow diverter was released in the distal end of the aneurysm. **(B)** The second flow diverter was telescoped from the distal to the proximal. **(C)** Angiography after release of the two flow diverters showed retention of contrast agent in the aneurysm. The parent artery was patent. **(D)** Postoperative Dyna-CT showed that the two flow diverters were satisfactorily open.

The first 5.5 × 45 mm Tubridge flow diverter (Shanghai MicroPort, China) was delivered to the head of the aneurysm via the microcatheter. The distal end of the first flow diverter was opened by pulling out the microcatheter, and the location of the distal end of the first flow diverter was determined. The first flow diverter was then slowly released in a push-based manner. After the first flow diverter was totally released, the microcatheter was conveyed to the distal end of the first flow diverter under the guidance of the flow diverter push guidewire. The second Tubridge flow diverter (6.0 × 45 mm) was delivered and opened inside the first, and then released slowly by combining a push and tension-reduction technique. The two flow diverters overlapped by ~25 mm (Figures 3A, B).

The angiography performed after the release of the flow diverter showed that the contrast agent was obviously retained in the aneurysm and the parent artery was patent [O'Kelly-Marotta (OKM)] grade B (5) (Figure 3C). Dyna computed tomography showed that the patency of the two flow diverters was satisfactory, in good agreement with the parent artery, and the entire lesion was covered (Figure 3D).

**Figure 4:**
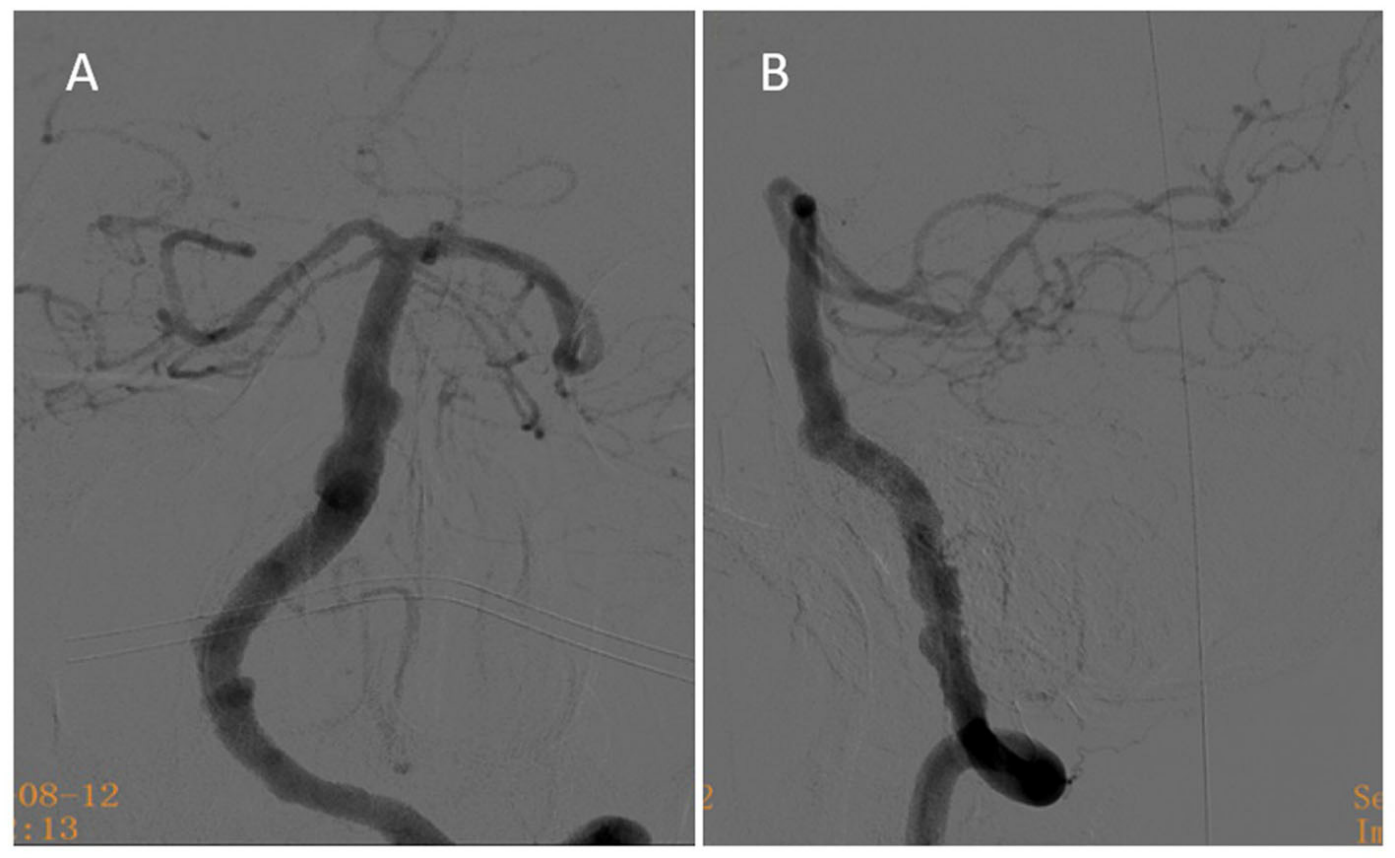
Six-month follow-up angiography. **(A)** At the 6-month follow-up, anteroposterior angiography showed that the aneurysm was nearly completely occluded. **(B)** At the 6-month follow-up, lateral angiography reveals the aneurysm was nearly completely occluded.

There were no signs of cerebral infarction after the operation, and the patient recovered well. He had no new symptoms after discharge and continued to receive dual antiplatelet aggregation therapy for 6 months. Six months after the operation, the follow-up digital subtraction angiography showed that the aneurysm was almost completely occluded, with OKM grade C (Figure 4). The antiplatelet strategy was then changed to aspirin (100 mg, 1×/d) with clinical follow-up.

## Discussion

Numerous clinical studies have reported the safety and efficacy of flow diversion in treating large intracranial and dissecting aneurysms (1, 6, 7). In recent years, flow diversion to treat vertebrobasilar aneurysms has also been applied, although off-label (8, 9). Few studies have evaluated flow diverter telescoping techniques for treating vertebrobasilar aneurysms. Yet, these techniques offer two advantages. First, multiple telescoping flow diverters to treat aneurysm of the neck effect significantly benefit blood flow changes, and promote thrombopoiesis in the aneurysm. Secondly, for large or dissected aneurysms, a single flow diverter is insufficient to cover the diseased vessels; for long-term efficacy, telescoping is required to completely cover the proximal and distal ends of the aneurysm (10).

The flow diverters now commonly used in China include the pipeline embolization device (PED) and Tubridge. The latter is longer; the maximum length is 45 mm. However, the Tubridge is weaker radially and more highly porous compared with the PED, larger aperture in design can reduce the incidence of perforator occlusion events. This is why the Tubridge flow diverter was chosen for the present case.

Dissecting aneurysms in the vertebrobasilar artery have a complex neurovascular anatomy (11). Placement of flow diverters inevitably results in coverage of basilar artery branches and perforators, the occlusion of perforator may be the most common cause of ischemia events (12). Having a perforators of the basilar artery occluded after FD implantation may occur by 2 mechanisms: the profile of the FD mechanically blocks the orifice of the perforators; or tiny thrombi form on the surface of the FD, which are then carried downstream by flowing blood causing perforators embolism. The smallest branching arteries of the basilar trunk are the perforators, which tend to have a diameter in the range of 80 to 940 μm, with a mean value of 400 μm (13). The Tubridge FD is a self-expanding device, it is composed of 46–62 nickel–titanium alloy microfilaments of 35 μm, and the pore size varies between 1,100 and 2,500 μm, depending on the final FD morphology and selection of the proper size adapted to the vessel diameter. The large-size Tubridge (>3.5 mm), which was mostly used in the posterior circulation, has less decreases the shortening rate after its full opening and offers lower pore attenuation (14). Tubridge FD has larger aperture in design, thus decreases the risk of mechanically blocks the perforators. In addition, in the present case, the overlapping area of the two FDs was selected at the proximal segment of the basilar artery with relatively few perforators to increased security.

Rigorous adequate standardized antiplatelet therapy (with reference to the thromboelastogram) can effectively reduce perforators thrombi embolic events. In the present case, the patient had no ischemic events occurred perioperatively. Rigorous testing of platelet function and subsequent regimen adjustments were crucial to minimize the risk of ischemic complications. Despite this, implantation of multiple FDs should be prudent in vertebrobasilar dissecting aneurysm, which helped to reduce thromboembolic complications.

In our patient, the flow diverter was deployed from the aneurysmal distal to the proximal, which is preferable to proximal-to-distal. Firstly, after the release of the first flow diverter, the re-superselection of the microcatheter can be conducted more easily in the channel of the first flow diverter. Secondly, when telescoping from the distal to the proximal aneurism (with reference to the heart), the first flow diverter and the pushing guidewire can be used to support the microcatheter. Compared with telescoping from proximal to distal, the guidewire support is longer, which can support the microcatheter more stably for re-superselection. In addition, it is necessary to ensure that the first flow diverter is firmly anchored in the distal parent artery through distal-to-proximal telescoping.

In this patient, the first flow diverter was anchored about 10 mm in the distal parent artery. A sufficient anchoring length at the distal end is especially important to ensure the stability of the distal flow diverter (15). Studies have confirmed that healing of the aneurysm is promoted when the flow diverter is attached to the wall of the tube after it is implanted. However, attachment is also associated with thrombosis and long-term stenosis and occlusion in the flow diverter itself (16). The telescoping technique may lead to poor adhesion of the flow diverter wall, requiring further treatment.

In the present case, a J-tip guidewire dilation in the flow diverter was performed after the Tubrige implantation to promote adherence of the flow diverter. The present case shows that vertebrobasilar dissecting aneurysm can be treated using telescoping flow diverters. However, we await the long-term results.

## Conclusions

This report is evidence that vertebrobasilar dissecting aneurysm can be successfully treated with a telescoping flow diverter technique with two flow diverters, and the treatment is feasible for this type of aneurysm. A prospective randomized study is needed to strengthen the evidence and determine the best conditions for applying flow diverter technology.

## Data availability statement

The raw data supporting the conclusions of this article will be made available by the authors, without undue reservation.

## Ethics statement

The studies involving human participants were reviewed and approved by First Affiliated Hospital of Dalian Medical University. The patients/participants provided their written informed consent to participate in this study. Written informed consent was obtained from the participant/patient(s) for the publication of this case report.

## Author contributions

M-YW and FW performed most of the investigation, data analysis, and wrote the manuscript. Y-SL, X-BA, and TP contributed to interpretation of the data and analyses. All authors have read and approved the manuscript.
